# ‘Everything turned upside down’: A thematic analysis of adolescents’ experiences of everyday life during COVID-19 restrictions

**DOI:** 10.1177/14034948231152272

**Published:** 2023-02-10

**Authors:** Line Indrevoll Stänicke, Pauline Olin Kurseth, Mona Bekkhus

**Affiliations:** 1 Department of Psychology, University of Oslo, Norway; 2 Lovisenberg Hospital, Nic Waals Institute, Norway; 3 Promenta Research Centre, Department of Psychology, University of Oslo, Norway

**Keywords:** Adolescence, COVID-19 pandemic, isolation, loneliness, mental health, peer interaction

## Abstract

**Background:**

At the beginning of the COVID-19 pandemic, people were encouraged to practice social distancing, and schools and leisure venues closed.

**Aims:**

We aimed to explore the everyday experiences of Norwegian adolescents during COVID-19 restrictions, when in-person contact with peers was severely limited.

**Methods:**

A total of 622 high-school students (16–18 years of age) replied to an online survey containing open-ended questions about the changes they experienced in everyday life during the first three months of the pandemic.

**Results:**

Reflexive thematic analysis resulted in four themes: (a) ‘Everyday life turned upside down – everything is on hold’; (b) ‘Alone with my thoughts – new concerns’; (c) ‘A loss of social life – a fear of wasting important time’; and (d) ‘Gratitude – new perspectives in life’. The results are discussed according to mental-health concerns and psychological developmental challenges during adolescence, such as social exploration of roles with peers, autonomy and identity formation during a crisis.

**Conclusions:**

**The results underline individual variations and positive experiences among adolescents during the COVID-19 pandemic, despite social restrictions. Still, the lack of in-person contact with friends is related to an increased experience of loneliness and mental-health concerns for many adolescents.**

## Introduction

The COVID-19 pandemic led to restrictions in many countries, involving social distancing and home schooling, and significant limitations on social contact. Social interaction with others is a basic human need, and engagement with peers plays a special role during adolescence [1]. Peer interaction enables social role and identity exploration and is based on adolescents’ own choice of companions compared to the interaction children have with their parents [2]. The outer boundaries represented by the caregivers become integrated and internalised, which increases ‘autonomy’ – a capacity to express your own meaning, to say no or to highlight important aspects of your opinion as different from others, even to an authority [3]. Peers play an important role in the exploration of closeness, intimacy and trust [4]. The capacity for mentalisation – to understand their own and others’ behaviour in terms of mental states, feelings and thoughts – develops during adolescence and increases capacity for affect regulation and a more stable self-identity [5]. Psychological development during adolescence is part of a life-long dialectic oscillation between relatedness and self-definition [6]. However, for adolescents in Norway and many other countries, the restrictions involving social distancing imposed during the first months of the COVID-19 pandemic led to a significant reduction in adolescents’ social contact with their peers. Although these changes were considered necessary to minimise viral transmission, little is understood about how these changes were experienced by adolescents from their own perspective.

Adolescence is a transitional life period, including cognitive, biological, psychological and social changes [1]. Many mental-health problems typically arise for the first time during adolescence and are exacerbated by overall increases in stress. Thus, social restrictions during the pandemic may have put youths at particular risk for developing mental-health problems [7]. For example, in their study, Orben et al. [8] found that physical distancing might have had a disproportionate effect on an age group for whom peer interaction is a vital aspect of development. Even though older people were at greatest risk of infection, those who were younger had increased risk of loneliness and symptoms of anxiety and depression [9]. Thus, in the context of the pandemic, adolescents may be at particular risk because of the reduced in-person contact with other peers [10], disruption in daily routines [11] and increased use of digital media [12].

To date, several cross-sectional studies have found an increase in mental-health problems, such as anxiety and depression, among adults [13] and adolescents [14] during the COVID-19 pandemic. In a cross-sectional study from China, mental-health difficulties increased more during the pandemic than prior to the pandemic [7]. Several studies further suggest that there has been an increase in symptoms of depression [15], anxiety [16] and emotional problems [17] during the pandemic. Similarly, longitudinal studies have reported an increase in mental-health disorders in the context of the pandemic. For example, a longitudinal study from Australia [18] found a significant increase in symptoms of depression and anxiety and a significant decrease in life satisfaction. Magson et al. followed 248 adolescents, 13–16 years of age, from before the pandemic to two months following implementation of the restrictions. Their findings suggested that the adolescents were more concerned about the imposed restrictions than they were about the virus. Another study reported a reduction in life satisfaction among adolescents early in the pandemic [19]. However, not all longitudinal studies found an increase in mental-health problems. In a Norwegian study, Hafstad et al. [20] reported only a marginal increase in mental-health problems in adolescents, which was related to their increasing age rather than the context of the pandemic. Previous findings from the current study, however, showed that higher levels of anxiety and depression were associated with less in-person contact with friends and spending more time on social media [21]. Although some findings on mental-health problems are mixed, the restrictions imposed during the pandemic may have put adolescents at increased risk for mental-health problems, and the consequences may have been especially hard on those with ongoing mental-health difficulties [22], such as those who self-harm and those who are more socially distanced [23]. In addition, a recent review reported an increase in social and risky behaviour problems among adolescents during the pandemic [14].

However, these studies do not add to our understanding of adolescents’ subjective experience of their everyday life in the context of the COVID-19 pandemic. Only a handful of qualitative studies have been conducted. Fitzpatrik et al. [24] found a high level of perceived need for mental-health services for both children and adolescents, as well as their parents. Other qualitative studies in Spain and Italy during the first period of lockdown have highlighted how children had mixed feelings about the restrictions – joy and relaxation about being at home with their family, but also fear, nervousness, loneliness, sadness and anger [25–27]. In a study in Ireland, O’Sullivan et al. [28] highlighted that the impact of the severe restrictions increased mental-health problems in families with vulnerable children. In adolescents, research findings point to challenges related to school [29] and increased loneliness because of the lack of in-person contact [30]. Staying at home was experienced as both a limitation and also a possibility to discover oneself and rediscover one’s family [31].

Although there have been a growing number of studies on the pandemic’s impact on adolescents, so far, few have asked adolescents in depth about their own perspectives with open questions. Often, adolescents’ perspectives on mental-health problems or consequences of the crises have been collected with questionnaires with response options. Thus, there is a need for increased knowledge about how adolescents experienced the pandemic more openly. Qualitative methods emphasise an exploration of a phenomenon to find variation and commonalities in the participants’ understandings and meaning making. In this study, we add to the current literature by including qualitative data and an in-depth exploration on adolescents’ experience of everyday life during the pandemic while adapting to social restrictions and new routines. We aimed to examine how adolescents experienced the changes in their everyday lives during the first weeks of the COVID-19 pandemic in order to obtain a more in-depth understanding of how the COVID-19 pandemic was experienced in terms of well-being and mental-health concerns in the early part of the pandemic. The findings are discussed in the context of some of the developmental challenges during adolescence, such as social exploration with peers for evolving autonomy and identity formation at this transforming period of life [1–4]. Even though the COVID-19 crisis may be over for the time being, this knowledge can inform clinicians, parents and decision makers on adolescents’ struggles during a crisis.

## Methods

### Participants and procedure

Participants were part of a cross-sectional study and were recruited via advertisements on the social media platform Facebook and on 10 high-school webpages in two urban counties. Recruitment was initiated on 1 April 2020, only two weeks after schools and social arenas were closed across Norway [21] and lasted until 23 April. We included participants who were 16–18 years of age and currently in school (*N*=622, 76.8% girls). Participants answered an online questionnaire containing well-known and validated close-ended questions about mental health, social media use and friendship (blinded for review), as well as three qualitative open-ended questions concerning their experience of changes in everyday life after lockdown. Before recruitment started, the survey was piloted among a small group of adolescents (*N*=10), and a few questions about social media use were adjusted for clarity before recruitment started. Of the 930 adolescents who received a link to the online questionnaire via text message, 709 (77%) completed the questionnaire, 87 of whom were excluded because they were ⩾19 years of age and/or were no longer in high school. The Regional Committee for Medical Research (REK: 124909), and the Data Inspectorate approved the study.

### The survey

The present study used the qualitative information from three open questions: (1) What were the biggest changes in your everyday life after the start of the COVID-19 pandemic? (2) What were the biggest negative changes in your everyday life? (3) Are there any changes that have been positive?

The participants submitted their responses to the open questions in the Norwegian language (see also data analysis), and all participants provided responses to the three open-ended questions, (i.e. there were no missing responses). The survey captured the first wave of the COVID-19 crisis in Norway and was conducted in a period with a low rate of infection in society. On average, participants answered the survey 30 days (range 19–56 days, *SD*=7 days) after the intrusive restrictions were implemented on 13 March.

### Qualitative research team

The qualitative research team consisted of all three authors. During the data analysis, we sought trustworthiness and methodological integrity by keeping the analysis transparent and close to the participants’ quotations [32]. We attempted to increase reflexivity by noting ideas and thoughts throughout the data analysis (‘reflective noting’). Although the researchers represented different theoretical and clinical backgrounds (neurocognitive psychology, psychodynamic and existential therapy), all were engaged in developmental psychology and research on adolescents. The results were discussed with a multidisciplinary research group (Promenta research group). In this way, we sought credibility so that other researchers would find the results meaningful [32].

In the present study, the epistemological position of critical realism informed our effort to discover how adolescents experienced changes in their everyday lives during the pandemic [33]. Within a critical realistic position, research aims to disclose reality, but at the same time, we as researchers cannot have an unmediated take on reality. The results are not a ‘direct view’ of the adolescents’ experiences but rather are labelled and represented by the informants and further interpreted by us as a research team.

### Data analysis

Reflexive thematic analysis (RTA) [34,35] was chosen to investigate the qualitative data from the open questions in the survey. RTA is useful for understanding participants’ subjective experiences of a phenomenon and to identify and interpret patterns in their meaning making of their everyday life, labelled as ‘themes’ and ‘sub-themes’ [35]. We used an inductive approach, allowing the data to inform the themes. Primarily, the themes were analysed based on the semantic content and were developed as close to the data as possible. Still, RTA highlights the researchers’ own contributions to an active and generative process and views research as a subjective process where the researchers actively contribute to the generation of themes. The researchers’ background in developmental psychology and clinical knowledge made us attuned to the participants’ responses concerning developmental challenges in adolescence and how differently they experienced and coped with the changes related to their familial and social context.

The data analysis consisted of six steps [34,35]. First, the informants’ answers were transferred to a spreadsheet, which made it possible to read each informant’s answer to every open question in the questionnaire. In this way, the researchers familiarised themselves with the data by reading and rereading the data set to explore the breadth and depth of the data material. Not all participants answered all questions, and the responses varied in length from 0 to 261 words per open question. We removed four bizarre answers because they contained no content related to the questions being asked in the survey.

Second, the researchers began the coding process by systematically and thoroughly generating initial ‘codes’ or short labels for relevant, interesting and meaningful parts and features of the data material close to the informants’ descriptions of their experience. The second author led the coding process and discussed the codes with the other authors. Thereafter, the first and second authors organised the codes into a general ‘groups of codes’. Some codes were dismissed, while others were grouped together or split apart. All groups of codes with some especially rich quotations were collected into a new working file to provide a condensed overview of the patterns of experience and meaning making throughout the data set. All researchers discussed the analysis, and we noticed that the adolescents described their experiences in a polarised way. What some experienced as challenging and difficult were for others considered a relief or something pleasant or positive.

**Figure 1.**
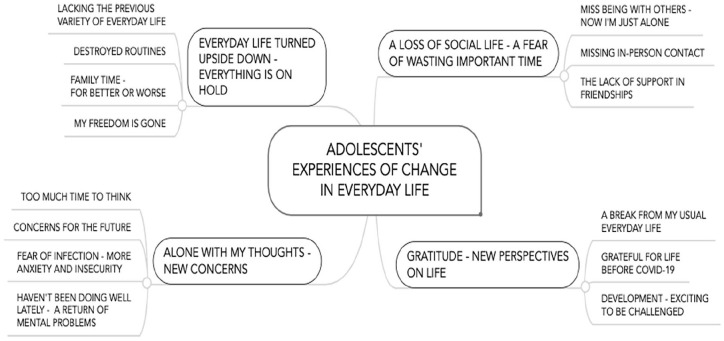
Hierarchy of themes and sub-themes.

Third, the first and second authors searched for themes by identifying patterns across the groups of codes. Fourth, we generated a hierarchy of sub-themes and encompassing themes, which represented the experiences of change in a thorough and accurate way (see Table I). The hierarchy of themes was reviewed by all researchers. We combined, split or relabelled the themes in ‘consensus meetings’ [32]. Fifth, the quotations, sub-themes and themes were translated into the English language, and careful attention was paid to capture the essence of the informants’ quotations. Themes and sub-themes were defined and named in the most meaningful and informative way (see Figure 1). The data analysis moved between different stages to generate meaning and stability in conceptualisation [32].

**Table I.** Example of different levels of analysis.

Theme	Subtheme	Group of codes	Quotations
A loss of social life – a fear of wasting important years	3.1 Miss being with others – now I’m just alone	Miss my friends	‘I’m a social person, and I’m not used to being away from my friends’.
		Being trapped at home	‘I was with people all the time. Now, I’m only on my own’.
		Feeling left out	‘[I’m] feeling isolated from social life. Even though nothing is happening, it bothers me’.

## Results

The analysis of the experience of change among high-school students during the COVID-19 pandemic resulted in four themes: (a) “Everyday life turned upside down – everything is on hold’, (b) ‘Alone in my own thoughts – new concerns’, (c) ‘Loss of social life – a fear of wasting important time’ and (d) ‘Gratitude – new perspectives in life’. In the following, we document and illustrate these four themes with associated sub-themes and selected quotations that are rich in details and capture nuances and divergent points in the data (see Table II). To increase transparency, we indicate the frequency of occurrence for each theme among the participants [36]: (a) many – 150 participants or more, (b) several – 50 to 150 participants, (c) some – 20 to 50 participants and (d) a few – 20 or fewer participants.

**Table II.** Overview of themes, sub-themes and group of codes.

Themes	Sub-themes	Group of codes
Theme 1: Everyday life turned upside down – everything is on hold	1.1. Lacking the previous variety of everyday life 1.2. Destroyed routines 1.3. Family time – for better or worse 1.4. Freedom is gone	1.1.1. From a hectic to a monotonous everyday life 1.1.2. Difficult to separate school and private life 1.2.1. No structures or frames for the day 1.2.2. Miss my teacher and the classroom 1.3.1. Too much time with my family 1.3.2. More connected with my family 1.4.1. Not allowed to do what I want 1.4.2. Not allowed to decide over my life
Theme 2: Alone with my thoughts – new concerns	2.1. Too much time to think 2.2. Concern for the future 2.3. Fear of infection – more anxiety and insecurity 2.4. Haven’t been doing well lately – a return of mental problems	2.1.1. A lot of time for reflection 2.1.2. Where do I belong? 2.2.1. Uncertainty 2.2.2. Nothing to look forward to 2.3.1. Fear of getting sick 2.3.2. Fear of infecting others 2.3.3. Fear in society 2.4.1. Afraid of having a relapse of mental problems 2.4.2. Overthinking 2.4.3. Boredom
Theme 3: A loss of social life – a fear of wasting important years	3.1. Miss being with others – now I’m just alone 3.2. Missing in-person contact 3.3. The lack of support in friendships	3.1.1. Miss my friends 3.1.2. Being trapped at home 3.1.3. Feeling left out 3.2.1. Miss face-to face encounters 3.2.2. Staying in touch with friends online 3.3.1. Can’t support my friends 3.3.2. Friends can’t support me
Theme 4: Gratitude – new perspectives in life	4.1. A break from my usual everyday life 4.2. Grateful for life before COVID-19 4.3. Development – exciting to be challenged	4.1.1. Less stressed 4.1.2. More time for interesting hobbies 4.1.3. Less pressure and less focus on appearance 4.2.1. Longing for everyday activities 4.3.1. Fun to experience coping 4.3.2. More time to find out what one wants in life

### Theme 1: Everyday life turned upside down – everything is on hold

The first theme, ‘Everyday life turned upside down – everything is on hold’, captures the experience of an overwhelming and chaotic change in everyday routines and family life. This theme consists of four sub-themes. The first sub-theme, ‘Lacking the previous variety of everyday life’, highlights how COVID-19 introduced a sudden change from a hectic into a monotonous everyday life for many participants – every day felt ‘the same’. Many of the adolescents experienced the change from normal school to home schooling as challenging and spoke of how difficult it was to separate their school life from their private life when being at home all day: ‘I don’t have any good routines anymore, either with school or with sleeping’. The second sub-theme, ‘Destroyed routines’, underlines how the structures and frames for their everyday life were gone, even broken: ‘I’ve lost my routines, and everyday life is messy’. Some adolescents reported how difficult it was to do all of their schoolwork at home in a new sphere without teachers and classmates. Some even considered school to be an important aspect of everyday life in terms of providing structure to their day and for handling mental-health concerns: ‘I am not able to take part in home school because of my depression and, therefore, I have a lot of spare time’. Their new everyday life seemed to create an experience of having to make new routines to replace the ones they had before the pandemic.

The third sub-theme, ‘Family time – for better or worse’, highlights how the students experienced spending more time at home with their family because schools were closed and because of the social restrictions. Some said they had ‘too much time together’ and that being at home so much ‘made them go crazy’ or ‘got on their nerves’. They described more quarrels and disagreements with their family members. Still, some reported that it was nice to spend more time with their family: ‘[I am] maybe more connected to my family, but there’s also more arguing’. Being at home may have created an experience of having little personal space. The fourth sub-theme, ‘Freedom is gone’, highlights the participants’ experience of being prevented from doing what they wanted and from making their own decisions. They could not meet friends and family members due to social distancing: ‘I feel like my freedom is gone. For example, I can’t see my boyfriend. I feel so lonely’. Some participants reported a lack of freedom to do exactly what they wanted when they wanted to do something: ‘I feel I don’t have the right to decide over my own life . . . everything is put on pause’. The first theme highlights how the participants missed their previous everyday routines and felt their life was on hold.

### Theme 2: Alone with my thoughts – new concerns

The second theme, ‘Alone with my thoughts – new concerns’, conveys how the COVID-19 pandemic brought new thoughts and worries for the participants. This theme consists of four sub-themes. The first sub-theme, ‘Too much time to think’, highlights how several informants reported a sense of suddenly having a lot of time to reflect upon small and big issues in their life: ‘I get more time to think, let the thoughts run wild. Thinking about life and wondering where’s my place and if I didn’t exist would anyone notice’. A few participants thought about where they belonged in the world. The second sub-theme, ‘Concerns for the future’, highlights an increased uncertainty about not knowing what will happen in the future, in both the short and long term: ‘Worrying about when all this is over’. Some reported that it was ‘difficult and scary to think ahead’ and asked themselves ‘what might happen next’, or they said that there was ‘nothing to look forward to’ because of cancelled events.

The third sub-theme, ‘Fear of infection – more anxiety and insecurity’, sheds light on the fear several had of becoming ill with the disease *and* their fear of infecting others: ‘I am afraid my mother and brother will have COVID-19 because they’re in the high-risk group’. They reported a fear of losing friends and family: ‘Anxiousness, panicking because I’m overthinking what can happen with friends and family’. Several described a sense of fear spreading through society or because of the news about the COVID-19 restrictions: ‘Everyone is in a kind of panic’. In addition, several participants described increased insecurity, difficulties concentrating, being restless, as well as a sense of losing control after the restrictions began. The fourth sub-theme, ‘Haven’t been doing well lately – a return of mental-health concerns’, conveys how some participants experienced symptoms of anxiety, panic attacks, depression, hopelessness, or meaninglessness, or reported that they were afraid they would have a relapse: ‘Since I’m inside a lot and alone, I feel very locked up. Since I can’t go out, I am in my own head a lot. This has led to my depression and anxiety coming back’. A few participants described overthinking because of boredom: ‘Boredom leads to overthinking, and I am afraid I’ll relapse with my mental health and [it will] get bad again’. In sum, the second theme sheds light on how restrictions during the pandemic gave rise to new concerns for many of the adolescents.

### Theme 3: A loss of social life – a fear of wasting important time

The third theme, ‘A loss of social life – a fear of wasting important time’, emphasises how the participants experienced changes in their social life and habits, and how they tried to adapt to these changes but still felt lonely. This theme consists of three subthemes. The first sub-theme, ‘Miss being with others – now I’m just alone’, conveys several participants’ experience of not being able to spend time with friends, peers and classmates. They described how they missed their friends and had to spend time in their own company, not because they wanted to but because of the restrictions. They described this change as challenging and unfamiliar: ‘I was with people all the time, now I’m only on my own’. Some described a feeling of being trapped at home, while others told stories about parents not allowing them to leave the house or meet up with friends. They underlined a frustration with being alone and left out of ‘ordinary’ social life: ‘[I’m] feeling isolated from social life even though nothing is happening, it bothers me’. They described a fear of wasting important time in their life: ‘I am losing valuable time’.

The second sub-theme, ‘Missing in-person contact’, emphasises how many of the participants missed face-to-face encounters with friends. This also included the lack of intimacy and not being able to experience physical contact, such as hugging, with friends on a regular basis: ‘I can no longer see my friends on a daily basis, and therefore I get much less physical contact than usual’. Some had more online contact with friends than usual, while others experienced online contact with friends difficult: ‘Since I’m a girl, I find it harder to keep in touch with friends regularly. That’s because we don’t sit and do gaming like the boys do’.

The third sub-theme, ‘The lack of support in friendships’, reflects how a few of the participants described an experience of not being able to be a friend in the way that they used to or wanted to be: ‘My friends are many hours away, and I can’t be there and support them when they really need it’. Some described how they miss getting support from friends and that the social restrictions influenced their mental health: ‘It’s hard for my mental health to not be as social as before, because I’m an extremely social person’. Spending time with friends and engaging in normal social activities, which are a significant part of adolescents’ life, were lost. Seen together, these sub-themes shed light on different aspects of how adolescents’ social life changed. Even though they knew that no one had a lot of social contact, they experienced a fear of missing out on something or wasting an important period in their life.

### Theme 4: Gratitude – new perspectives in life

The fourth theme, ‘Gratitude – new perspectives in life’, captures the positive experiences, as well as the learning and growth adolescents experienced during the pandemic, despite the considerable changes and losses that occurred during this difficult time. This theme consists of four sub-themes. The first sub-theme, ‘A break from my usual everyday life’, underlines how the changes and restrictions during the pandemic brought more spare time in their everyday life. Several participants described how the pandemic brought a pause from their usual hectic life. They felt calmer and less stressed: ‘It can be pleasant to have nothing to do. I’m not used to it, and so it’s a little nice sometimes to have time to relax’. Further, several participants described more time to do new things and engage in leisure activities, interesting hobbies or relationships than before: ‘[I have] more time to read borrowed books, be with family, reflect on different things, spend more time out walking’. They had an experience of gaining something, having more time for creativity and more play. A few participants emphasised less pressure on appearance in dressing, hair and make-up when at home: ‘There’s less pressure on things you notice in school and such. As what clothes to wear, if you wear make-up or have spent time in the morning to fix your hair etc. There’s no focus on appearance’. They found it relieving and did not experience the same social demands and strains to ‘fit in’.

The second sub-theme, ‘Grateful for life before COVID-19’, conveys how some missed school:

In general, I have always loved going to school, but I still think I have gained a slightly new perspective on going to school. I miss sitting in classrooms, meeting friends in the hallways, picking up books in the lockers, etc. I even miss taking the bus! So, I think everything that happens now will give me a different perspective and a greater appreciation for everyday things when this is over.

Many longed for common events in their everyday life and appreciated these things now that they no longer had the opportunity to do them.

The third sub-theme, ‘Development – exciting to be challenged’, conveyed how the COVID-19 pandemic and social distancing became an opportunity for new experiences. For a few participants, it was exciting to experience coping: ‘More experience of coping with mental challenges. It’s fun to cope with a new and different situation’. It was exciting to discover their own strengths. Some participants described more time to find out what they want in life and more time to think about wishes for the future, career choices and new activities: ‘I feel I have more time for myself and to find out what is actually important in this life’. A time of struggle and hardship along with more time for reflection during the pandemic brought an opportunity for finding a direction in life. The shutdown caused by COVID-19 seemed to slow the pace of everyday life and create an opportunity for growth and new perspectives.

## Discussion

In the current study, we examined how adolescents experienced changes in their life after the first lockdown due to the COVID-19 pandemic. The main findings show that adolescents experienced their everyday life being turned upside down. They felt less freedom and alone with their thoughts, and they experienced a loss of social life during an important period of their life. Still, many also felt gratitude and gained new perspectives in life.

Previous findings from studies on adolescents’ mental health during the pandemic are mixed. Some studies report increased loneliness, anxiety or depression among adolescents [9], and other studies report that the social restrictions and home schooling did not lead to an increase in mental illness or behavioural problems [20]. These studies, however, only include data from questionnaires with predetermined answers. In the present study, adolescents answered the open questions about pandemic-related changes, providing an opportunity to describe experiences and meaning making of the changes from their own perspective. These first-hand experiences are important contributions to the field because they add nuances and may increase our understanding of the mixed results across several quantitative studies. In what follows, we discuss how the participants’ descriptions of mental-health concerns may provide nuance results with quantitative data. Further, we aim to understand the participants’ experiences of the pandemic in relation to developmental challenges during adolescence, such as social exploration of roles to evolve autonomy and identity formation during a crisis.

### Mental health concerns

Some quantitative studies [14–17] and qualitative studies [29,30,37] have described how adolescents have struggled with both school and mental-health issues during the COVID-19 pandemic. In our study, several of the adolescents described difficult thoughts or worries, such as a feeling of being alone with their thoughts, having too much time to think and concerns for the future. Many of the participants described mental-health concerns, such as ‘feelings of loneliness’, ‘anxiety’ or ‘depressive thoughts’, which are an important expression of how these adolescents felt at the time. However, we do not have follow-up data, and therefore we do not know whether these feelings of anxiety and depression persisted. The adolescents’ descriptions, however, do not necessarily reflect clinical levels of anxiety or depression. Their report may be representing an experience of mental-health concerns in the moment. Still, it is important to underline that a restricted social and physical context may be experienced as challenging and overwhelming. Therefore, their expression of being ‘anxious’ or ‘depressed’ should be acknowledged. This is particularly worrisome, as adolescence is a vulnerable time, with a peak in mental-health disorders [1]. Thus, an increase in anxious feelings and depressed mood may set some adolescents on a vulnerable path to anxiety and depressive disorders in adulthood. In Norway, for example, there has been a moderate increase in referrals to public mental-health units in general and a significant increase (up to 30%) in referrals for severe symptoms, such as psychosis, eating disorders, self-harm and suicide ideation – especially among children and adolescents – during some phases of the pandemic [38]. Even though the adolescents’ descriptions of mental-health concerns do not provide information on clinical symptoms and the results cannot give the long-term effects of the restrictions, the sudden changes and lack of in-person contact with friends may be related to an increased experience of loneliness and mental-health concerns for many of the adolescents. During adolescence, emotions are intensely experienced, and the cognitive capacity to handle and integrate experiences may not be sufficiently developed [39]. Participants in our study described having fears of getting sick or infecting others, and how they felt influenced by society’s fears about COVID-19. The comprehensive restrictions, especially at the beginning of the pandemic, may have been difficult to understand for some children and young adolescents because of the seldom-visible concrete danger of the disease. It is further possible that living with an invisible danger and social and physical restrictions over time may increase mental-health problems during this time.

### Exposure to change

During the COVID-19 pandemic, a whole generation of adolescents experienced changes in their everyday life. In our study, they described everything being turned upside down, a lack of freedom and life being on hold. The changes included less activity, less structure and routines, no borders between school and home and, for some, social isolation. During adolescence, a developmental challenge is to find your own solutions and to trust your own choices towards increased independency and separation [1,2]. Social and physical exploration may be a way to test strategies and problem solving to find autonomy [3]. The pandemic may have hindered adolescents in social exploration, which is important to increase autonomy. Even though they spent more time with their family, adolescents’ primary orientation towards peers may evoke a feeling of being alone. Contact with peers is of vital importance for being seen and validated in the process of testing norms and social roles, in finding identity formation and in the process of finding their own solutions and exploring interests.

Moreover, adolescents live in a social context, and the restrictions may have influenced adolescents in different ways according to individual and contextual risk factors, as well as protective factors. For example, those who had a hard time at school with bullying and social problems with peers before the pandemic could possibly have felt relief at being able to spend more time at home and to have more time with parents and siblings. Maybe they felt less challenged and safer. Others who struggled with mental-health concerns or problems at home before the pandemic may have missed the structure provided at school. Not being seen by a teacher and the lack of contact with friends could potentially have increased mental-health concerns. In sum, the findings highlight the variety in adolescents’ experiences of change during the pandemic: some enjoyed more time at home with their family, but others felt the physical and social restrictions as hindering their exploration and freedom.

Our study further underlines how adolescents described an achievement of new perspectives in life caused by the pandemic. The break from their usual everyday life may have enabled a pause and some time for reflection, and the chance to notice and be grateful for the good things in their everyday life, which are otherwise easy to take for granted. Some expressed that the challenges were exciting. Thus, some adolescents may adjust to a new context quite quickly. One study showed that adolescents’ perceptions of change in their relationship quality during the pandemic were heterogenous, and higher perceived change and instability were associated with poorer psychosocial functioning [40]. The variety in experience of the pandemic is important to relate to individual and social and contextual resources and risks. Additional support may be needed for those experiencing and perceiving the most difficult challenges or greatest change in their everyday life.

### Lack of friends and peers – a fear of missing out even though nothing happened

For long periods during the pandemic, adolescents were at home all day, with their only social contact with peers being through digital channels and arenas. Increased experiences of loneliness and social isolation among adolescents during the pandemic have been reported in several studies [21,30]. In our study, the participants described a loss of social life. Even though nothing was happening, they still felt as if they were ‘missing out’ on something important. It was as if their life was on hold. Especially during adolescence, contact with friends and peers is important for social and psychological exploration and for testing of social roles and boundaries in the process of identity formation. Normally, social isolation may be understood as a risk factor for mental illness [1]. During the pandemic, the restrictions could be described as an experiment with a whole generation. Adolescents lost social contact during a developmental period where social exploration is at its peak. However, many of the study participants stayed in touch with their friends on social media. Adolescents in developed countries use social media platforms (e.g. Snapchat, Instagram, TikTok, Facebook and Messenger) to communicate by chatting via text, to share pictures or videos and to search for information [42]. Consequently, social media may have created spaces for socialisation with old and new friends during the pandemic when in-person contact was limited. Thus, in this context, social media may be understood as an extended arena for peers [21]. Indeed, during the pandemic, adolescents reported an increase in the use of social media and gaming [41]. It is possible that digital communication and interaction can compensate for the lack of in-person contact with friends and peers to some degree, enabling the maintenance of close interpersonal relationships and facilitating the formation of close and meaningful new relationships [43]. For example, boyd suggests that even if the communication is not physically face-to-face, adolescents communicate with friends on social media similarly to how they communicate in person [44]. In this way, social media may have played a pivotal role for socialisation during the pandemic for many adolescents, and as such, digital communication may decrease feelings of loneliness and increase well-being. Thus, for some, it may be easier to connect with others or to communicate about personal content online. For others, it may be more difficult to express and share personal experiences without face-to-face contact. Adolescents are especially oriented towards others’ facial expressions and emotional signals [45]. Thus, in-person contact may include more information on eye contact, feelings, body language and non-verbal cues [46], and for some adolescents, a reduction in social information may have negative consequences. Some may even feel rejected or dismissed because of the lack of expected responses in online communication. The possibility of sharing good and difficult experiences and exploring roles and borders with friends and peers is important for developing mentalisation and an understanding of themselves and others [5]. Therefore, one might feel lonelier not only because of the lack of in-person contact but also because of the lack of information in the digital contact. Even though findings show that digital communication predicts higher quality friendships and greater well-being [47], our study highlights that adolescents’ online communication and interactions may differ according to their individual and social resources. However, girls were overrepresented in our study, and therefore our findings may limit how well the results represent young men’s experiences during the pandemic.

### Recommendations for public health initiatives

The implication of our findings suggests that closing schools and other social areas should be limited to a shorter period for adolescents. Digital health services, such as open chat functions with school social workers and health nurses, could be important to limit the mental-health burden during adolescence. Schools should increase social platforms for adolescents to interact and socialise during future pandemics.

### Strengths and limitations

One strength of this study is that the data collection was conducted just two to three weeks after the government closed schools and other social arenas. The adolescents were asked about their own perspective and feelings in this situation. Thus, the results of this survey may present a picture of the situation and illuminate adolescents’ experiences from a here-and-now perspective. It must nevertheless be emphasised that the results are presented as an image of a certain state during a time of urgency. Further data collection may be able to show longitudinal changes in adolescents’ experiences or long-term effects of social distancing and lockdown. Even though the sample size does not outweigh the quality of the data, the rather large sample may allow for the reporting of patterns across the data set [32].

The themes identified in this study are the result of an analysis of questions in the survey that focused on changes in everyday life and, in this way, presumed that there had been changes in everyday life. However, it is still very likely that the vast majority experienced changes to some degree, and this study explores *how* the participants experienced those changes. Further, the third question asked whether there had been any positive changes. This wording may have caused the participants to think about *either* positive or negative consequences and not openly describe their experiences. Still, the variety in experience of their everyday life was the overall focus of the analysis.

We used a convenience sample, and information about the family and socio-economic background of the participants was not collected. Also, girls were overrepresented in our sample, which may limit how well the results represent young men’s experiences during the COVID-19 pandemic. It is possible that this reflects our recruitment method (via Facebook), as girls use social media (i.e. Facebook) more than boys in general [48]. However, it may also be that Facebook is not the most active social media platform for this age group, although we also advertised the study on several schools’ websites. Further, the results represent a picture of the adolescents’ experiences in the context of a specific pandemic and cannot be generalised to adolescents’ experiences in another crisis. The results are closely related to the context, and the COVID-19 restrictions and consequences were different in Norway than in other countries. However, the concepts developed from this study may be discussed in relation to concepts and theory from other studies of how adolescents experienced the pandemic and may be discussed in relation to concepts and theory developed in another crisis.

Reflexive thematic analysis is an appropriate method for analysis seeking to understand experiences [34,35]. However, the current data used short textual answers to open-ended questions. Here, answers are taken at face value, and we therefore must assume that the adolescents’ written answers reflect their understanding and meaning making of their everyday life. Although these open-ended questions provided more in-depth knowledge about the adolescents’ experiences than survey questions with pre-categorised answers, the lack of in-depth interviews, which include facial gestures and non-verbal information, is a limitation of this study. However, in-person interviews were not possible during this time of the pandemic. A major strength of the current study was that we could target the adolescents in the very first weeks after the lockdown (which very few other studies were able to do). This would not have been possible if we were conducting online interviews during this early phase of the lockdown, as there was no access to research assistants or researchers who could organise and conduct online interviews.

## Conclusions

This study explored adolescents’ experience of changes during the first COVID-19 wave [21]. Reflexive thematic analyses of answers to open-ended questions showed that adolescents described everyday life as being turned upside down. They felt a lack of freedom, alone with their thoughts and a loss of social life. They were afraid of wasting important years of their life. Still, they felt gratitude and gained new perspectives in life. These findings highlight the variety of experiences among high-school students at the very beginning of the pandemic. The start of the pandemic in Norway reflected a very sudden lockdown, with much uncertainty. Thus, this study provides an insight into adolescents’ own perspectives and experiences during the acute phase of a crisis. Unfortunately, we do not have any follow-up data to examine long-term consequences. The results show how several of the adolescents described mental-health concerns, and this may suggest that additional support could have been helpful for some to handle the crisis. Further, adolescents’ experiences during the pandemic may be understood according to psychological developmental challenges, such as gaining autonomy and the importance of social exploration with friends and peers. The findings should be interpreted in light of the use of short textual answers to open-ended questions, even though the concepts may be analytically discussed in relation to concepts and theory from other studies with different samples and contexts. Finally, our study points to the need for qualitative studies reflecting in-depth understanding of adolescents’ own perspectives and capturing variability in their everyday experiences during the pandemic.
